# Context-Dependent Roles of the Plexin-B Family Across Neurological, Oncological, Immune and Cardiovascular Disorders: Mechanisms and Therapeutic Implications

**DOI:** 10.3390/life16091523

**Published:** 2026-09-14

**Authors:** Jianli Xu, Nannan Wang, Jun Lao, Renwen Zhang

**Affiliations:** College of Biology and Food Engineering, Jilin University of Chemical Technology, Jilin 132022, China

**Keywords:** plexin, neurological diseases, cancer, immune, angiogenesis

## Abstract

Plexin-B1, Plexin-B2, and Plexin-B3 are members of the Plexin-B receptor family, which engage distinct class IV semaphorins as major ligands: Plexin-B1 binds *SEMA4D*, Plexin-B2 interacts with *SEMA4C* and *SEMA4B*, and Plexin-B3 has been reported to bind HSPA5, while all three may participate in non-redundant ligand–receptor networks. Researchers have found receptors associated with neurological and malignant, immune-mediated, and cardiovascular diseases. However, this study strives to provide a comprehensive, cross-disciplinary synthesis of the context-specific functions of Plexin-B family members and to critically evaluate their emerging therapeutic potential across multiple disease domains. In a tumor- and context-dependent manner, Plexin-B1 and Plexin-B2 promote metastasis in glioblastoma, colorectal cancer, and triple-negative breast cancer (TNBC) through RhoA/Rac1-dependent signaling, while Plexin-B3 exhibits dual roles, acting as a tumor suppressor in TNBC under hypoxic conditions yet promoting motility in pancreatic cancer. Plexin-B regulates neuroinflammation in Alzheimer’s disease and T-cell regulation in asthma and other cardiovascular diseases via signals. This review positions the Plexin-B family as a promising yet functionally complex target for future precision medicine.

## Introduction

Plexins are receptors for semaphorins. While most are known to guide axons, recently, many have also been found to be involved in various diseases [1]. The four groups of receptor families are Plexin-A, Plexin-B, Plexin-C, and Plexin-D. The Plexin-A group includes A1–A4, and the Plexin-B group includes B1–B3. Plexin-C1 and Plexin-D1 are the only members of their respective subfamilies. There are a few types of semaphorins. Classes I and II occur in invertebrates, and vertebrate semaphorins belong to classes III–VII. Class III proteins are secreted, and the members of classes IV–VII are membrane-associated. Humans express seven class IV semaphorins, *SEMA4A*-*SEMA4G*, which are involved in autoimmune, neoplastic, and degenerative diseases, among others [2].

Plexin-A, -B, -C, and -D receptors promote neurogenesis and neuronal differentiation [3]. Among the Plexin-B family members, B1, B2, and B3 are single-pass transmembrane receptors that regulate neuronal genesis, neural-cell migration, and adult glial differentiation via semaphorin–plexin complexes [4,5]. Accurate Axon navigation requires coordinated signaling by semaphorins, netrins, slits, and ephrins, and alters the cytoskeleton during the movement of growth cones to their destinations. NRP1 is a component of the semaphorin–plexin pathway that supports neurite outgrowth, axon guidance, cell growth, and migration [6]. Suppression of Sema3A/NRP1 signaling improved hindlimb motor function and increased the number of motor neurons in the injured spinal cord in experiments [7,8,9].

**Figure 1:**
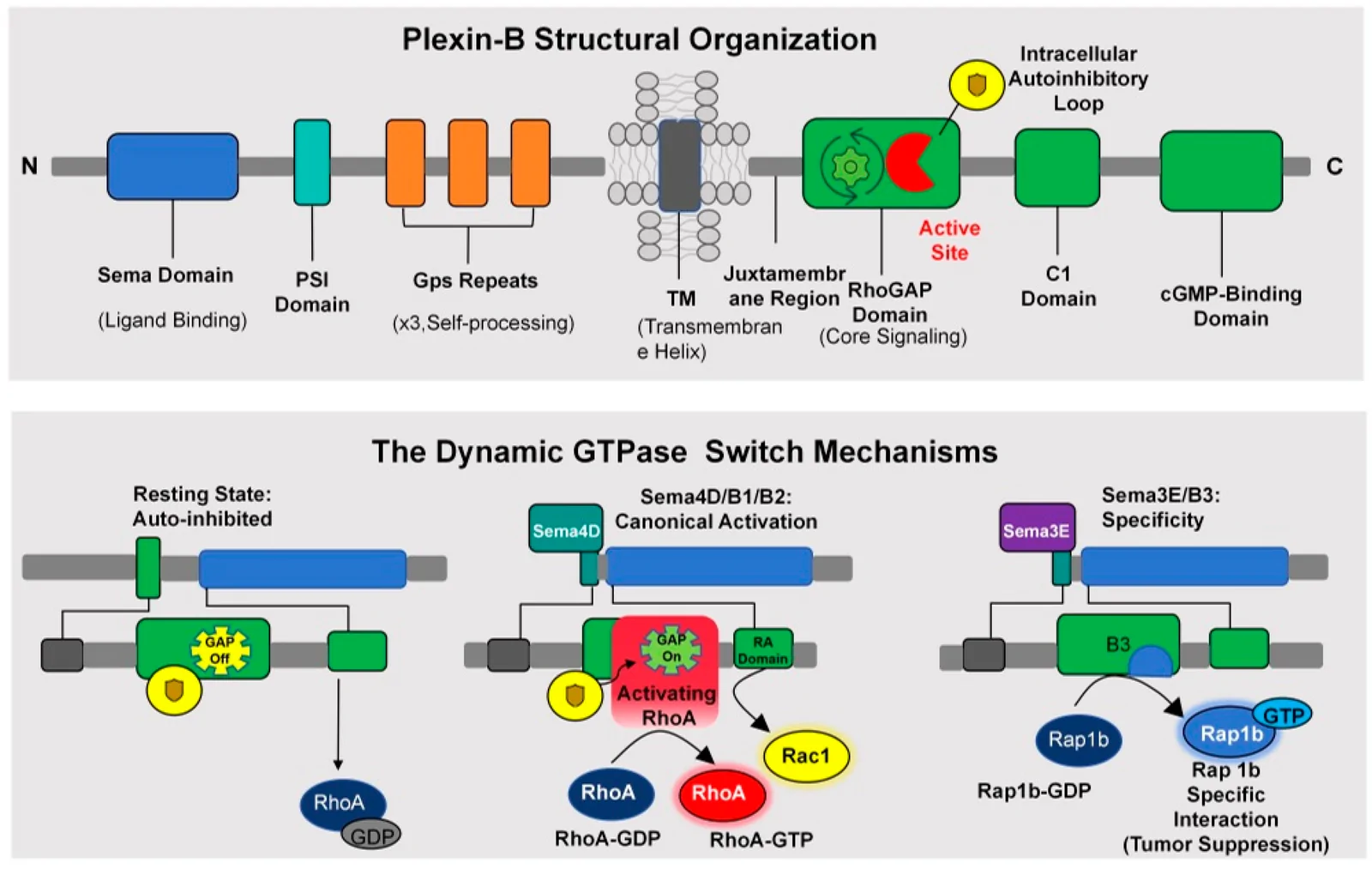
Plexin-B structure organization and the dynamic GTPase switch mechanisms.

A critical conceptual framework for this review is that Plexin-B1, B2, and B3 are non-interchangeable entities (Figure 1). Their expression patterns differ markedly: *PLXNB1* is widely expressed in astrocytes, endothelial cells, and immune cells [10,11,12]; *PLXNB2* is prominent in neural progenitors, macrophages, and tumor-associated stroma [13,14,15]; and *PLXNB3* has a more restricted distribution in oligodendrocyte progenitor cells (OPCs) and retinal ganglion cells [16,17]. Downstream, Plexin-B1 uniquely possesses R-Ras GAP activity in addition to RhoA activation [18]. Plexin-B2 preferentially signals through RhoA/ROCK and ID1/3 [19,20]. Plexin-B3 couples to MET and hypoxia-responsive pathways [4,21,22].

Recently, people have been exploring the molecular regulation, disease associations, and therapeutic strategies for plexin proteins, focusing on Plexin-B1 and Plexin-B2. TMEM260-dependent O-mannosylation promotes the maturation of Plexin-B2, and structural analysis of Plexin-B1 has identified an allosteric mode for signal transmission [23,24]. Circulating *PLXNB2* levels correlate with diabetic retinopathy risk [25], and rearrangement of the Plxnb1 genomic region has been associated with altered chromatin architecture in non-alcoholic fatty liver disease [26]. In preclinical models of postmenopausal osteoporosis and multiple sclerosis, an antagonist anti-Plexin-B1 antibody has been shown to produce a beneficial effect and is being further explored as a potential therapeutic target [27,28] (Table 1).

**Figure 2:**
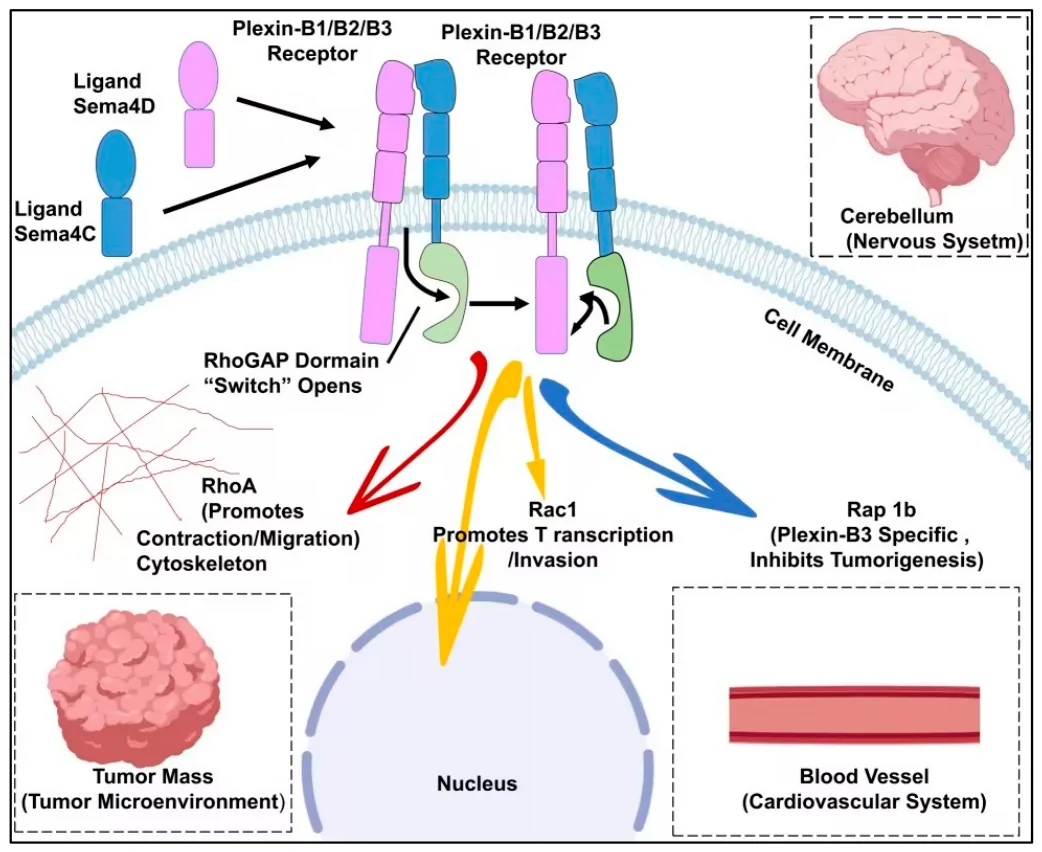
The integration of the neurological, oncological, immune, and cardiovascular dimensions of the Plexin-B family within a single translational framework. Elements of the cerebellum, cell membrane, tumor mass, and blood vessel were created with BioGDP.com.

**Figure 3:**
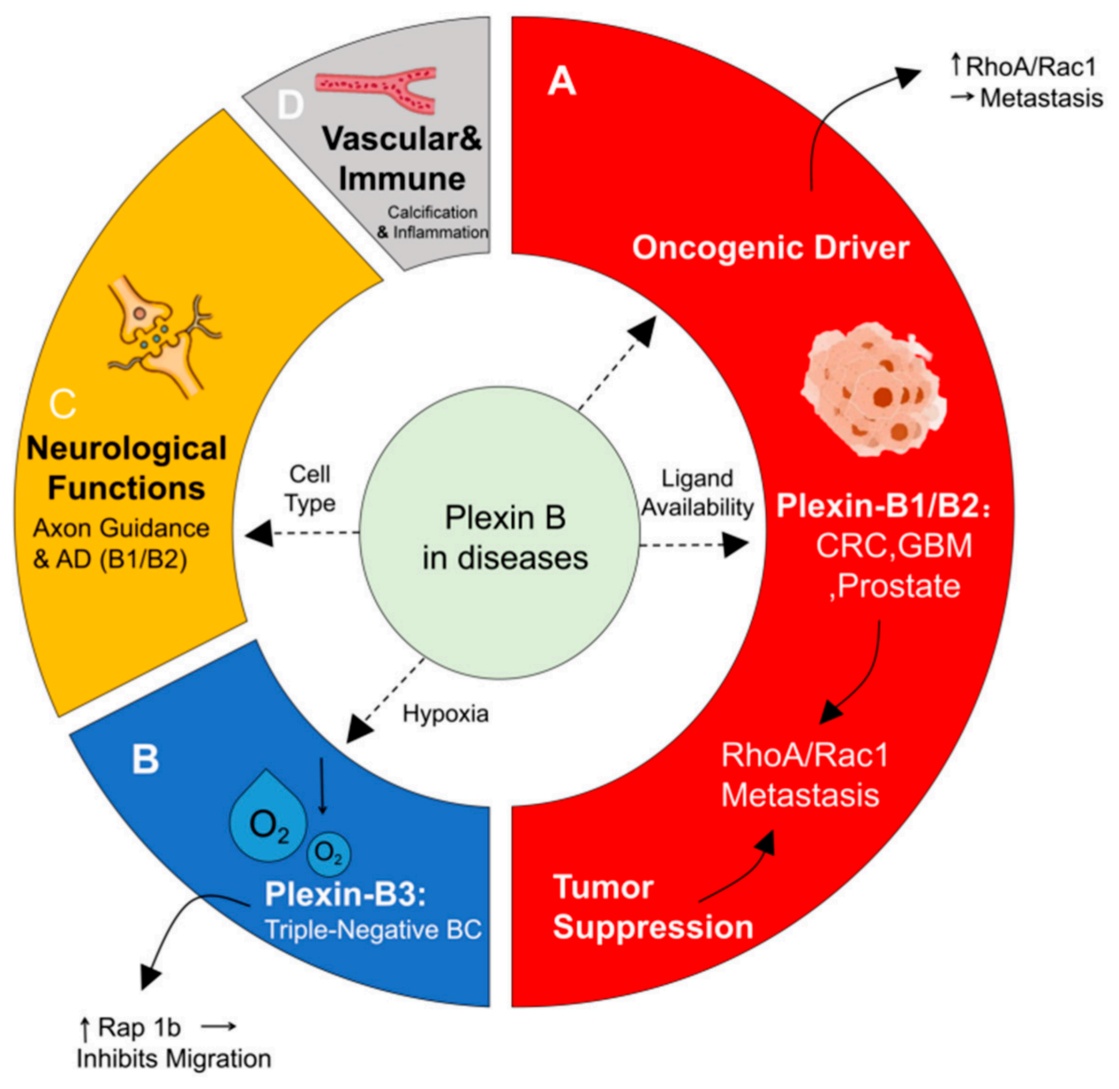
The Plexin-B family as a unified yet functionally divergent target in precision medicine, involved in cancer, immune disorders, and neurological and cardiovascular diseases. Elements of the tumor and vessel were created with BioGDP.com.

The first authoritative evaluations generally considered plexins from a single disciplinary angle. Khan and others focused on neural regulation and brain diseases [3]. Toledano and Neufeld [8] studied the problems of cancer cell proliferation and invasion, and Bica et al. [17] explored the function of semaphorins in malignancy. This review provides neurological, oncological, immune, and cardiovascular evidence in a single translational framework (Figure 2). By comparing the context-dependent mechanisms of the four areas and critically evaluating the new interventions, we can see that members of the Plexin-B family have different functions and therapeutic effects (Figure 3; Table 2).

**Plexin B protein in neurological diseases**

Plexin-B1, Plexin-B2, and Plexin-B3 influence neural development through complementary mechanisms. Plexin-B3 promotes retinal axons crossing the midline [29], while Plexin-B2 maintains cortical mechanical tension that prevents premature neuronal differentiation [30] and shows increased expression in relevant nervous-system settings [31]. Plexin-B1 transduces *SEMA4D* signals that remodel the astrocytic cytoskeleton [32]. Human genetic evidence further indicates that pathogenic *PLXNB3* variants can produce congenital heart defects together with neurodevelopmental impairment [33]. Thus, disruption of Plexin-B-dependent axon positioning, force sensing, or cytoskeletal organization may provide a mechanistic basis for neurodevelopmental disease and associated cardiac abnormalities.

Plexin-B1 is an important regulator of astrocytes and neuroimmune communication. Around amyloid plaques, it controls astrocyte–microglia spacing and thereby contributes to Alzheimer’s disease pathology; loosening this Plexin-B1-dependent cellular network may attenuate neuroinflammation [34,35]. Cerebrospinal fluid proteomics has also associated Plexin-B1 with bipolar I disorder [36], and astrocytic Plxnb1 is markedly elevated in PS19 tauopathy mice [37]. After spinal cord injury, the receptor helps preserve astrocyte morphology and membrane organization, aligns glial processes, corrals the lesion, and guides regenerating axons [10]. Plexin-B1 and Plexin-B2 separately participate in GABAergic synapse formation, potentially because their transmembrane regions confer different signaling properties [38].

For Alzheimer’s disease, Plexin-B1 controls astrocyte–microglia spacing around amyloid plaques, such that its loss loosens glial organization, reduces neuroinflammation, and promotes amyloid compaction [35]. Plexin-B3-positive OPCs naturally secrete amyloid-β in AD models, directly linking B3+ progenitors to plaque pathology [16]. Microglial Plxnb2 is elevated in tauopathy models, consistent with tau-dependent glial activation [37]. These observations suggest that Plexin-B1/B2/B3 influence AD through complementary routes: astrocyte–microglia spacing (B1), tau-associated glial reactivity (B2), and amyloid-β production by OPCs (B3). However, the causal direction remains unresolved; it is unclear whether these reflect primary drivers or adaptive responses to neurodegeneration.

A ligand mutant that still binds Plexin-B1 nevertheless fails to stabilize regulatory T cells in peripheral blood mononuclear cell cultures, illustrating the specificity of this pathway [39]. During peripheral nerve repair, Plexin-B1 directs Schwann cell positioning and coordinates Schwann cell–axon interactions [40]. Biomimetic LAsomes can exploit *SEMA4D*–Plexin-B1 interactions to traverse the blood–brain barrier before reaching homologous target cells [41]. Increased Plxnb1 expression has also been reported in ciprofloxacin-treated mice with tendinopathy [42]. Finally, acute Sema4D/Plexin-B1 stimulation can trigger inhibitory synapse assembly with sufficient temporal precision to permit analysis of individual synaptogenic events [43].

Plexin-B2 converges multiple neural and glial signaling programs. Plexin-B2, as a receptor for *SEMA4C*, could activate RhoA in combination with the ID1/3 transcriptional pathway [19]. At presynaptic terminals, binding to semaphorin-2b and to activated integrins facilitates presynaptic homeostatic potentiation [44]. Axonal extension also relies on tightly regulated endocytosis: Cdk5 and GSK3β phosphorylate endophilin, disrupting its interaction with dynamin-1 and CRMP4, thus inhibiting rapid internalization and synchronizing axon elongation, branching, and growth cone development in hippocampal neurons [45]. Microglial Plexin-B2 additionally responds to Sema4B and links immune signaling with glial behavior. This pathway has been implicated in stress responses and mechanisms governing susceptibility to schizophrenia [46]. Conditional loss of Plexin-B2 in myeloid cells [47] disorganizes macrophage positioning and extracellular matrix structure, destabilizes Schwann cells, misdirects axons, and delays functional recovery after injury [13]. Presynaptic Plexin-B2 in interneurons is also necessary for GABAergic synapses onto CA1 pyramidal neurons [38], while *SEMA4C*–Plexin-B2 signaling increases dendritic complexity and synapse density in the adult hippocampus through RhoA activation [48]. Plexin-B2 can serve as a receptor for angiogenin and thereby regulate cell survival and proliferation [49]; BDNF and vascular endothelial growth factor may engage this angiogenin/Plexin-B2 axis during spinal cord repair. In the cerebellum, *SEMA4C*-dependent migration requires Plexin-B2 [50], which also constrains the movement of granule neurons [51]. Semaphorin-4C, Plexin-B2, and neuropilin-1 therefore provide useful molecular markers and experimental entry points for studying neuronal growth and synaptic connectivity in cerebellar systems [27,52]. Following intracerebral hemorrhage, membrane-associated *SEMA4C* signaling can reduce neuronal apoptosis, whereas cleavage or delivery of soluble ligand may weaken or reverse this benefit [19]. Microglial Plxnb2 is elevated in tauopathy models, consistent with tau-dependent glial activation that can be modified by SORLA upregulation [37]. Finally, muscle atrophy is accompanied by reduced Sema4A; restoring the ligand limits dexamethasone-induced wasting and accelerates recovery through its functional receptor Plexin-B2 [53].

Plexin-B3 has a more confined neural distribution and is relatively abundant in adult oligodendrocyte progenitor cells. Extracellular HSPA5 binds to Plexin-B3 and regulates oligodendrocyte maturation and myelin production via Nkx2.8 and downstream effectors [54]. Plexin-B3-positive progenitors are also a source of amyloid-beta in vivo and may contribute to Alzheimer’s disease pathology [16]. Genetic and functional studies have also connected Plexin-B3 signaling with autism spectrum disorder [55].

Several mechanisms remain unknown. Although Plexin-B3-positive oligodendrocyte progenitors naturally secrete amyloid-β in Alzheimer’s disease models, it is unknown whether this activity causes pathology or is an adaptive response. Plexin-B2 signaling has also been associated with both vulnerability to and protection against stress in schizophrenia; thus, the neuropsychiatric effects may differ among various cell types or circuit environments. Plexin-B1 and Plexin-B2 can independently organize GABAergic synapses despite being ligands for the same receptor, but the molecular reasons for this receptor-specific behavior have not been identified.

**Plexin B protein in tumorigenesis**

Plexin-B receptors have many—and sometimes conflicting—functions in cancer. Plexin-B2 promotes hepatic colonization by colorectal, pancreatic, and melanoma cells through RAC1 activation [56]. The *PLXNB2* locus also generates lncFAL, which promotes FSP1, inhibits ferroptosis, and thus promotes the proliferation of hepatocellular carcinoma [57]. Plexin-B1 mutations are associated with altered invasiveness in prostate cancer [58]. Plexin-B3 is overexpressed in pancreatic cancer, and the loss of this receptor can disrupt actin organization and alter cell migration; thus, it is no longer tumor-suppressive [59]. Plexin-B2 knockdown increases the proliferation but reduces the migration of rhabdomyosarcoma cells [60]. Therefore, the same receptor family may promote or inhibit the development and spread of tumors; thus, both disease and the cellular environment must be considered in the development of therapy.

A key mechanistic distinction is that Plexin-B1 functions as both an R-Ras GTPase-activating protein (GAP) and a RhoA activator. The R-Ras GAP activity is facilitated by Rnd1 binding, which promotes inactivation of R-Ras and consequent cytoskeletal rearrangement. Simultaneously, Plexin-B1 recruits PDZ-RhoGEF/LARG through its C-terminal PDZ-binding motif, leading to RhoA activation and ROCK-dependent contractility. These are parallel, non-redundant signaling branches. Downstream pathways beyond RhoA/Rac1 include Pyk2-PI3K/AKT, MAPK/ERK, RhoGEF activity (PDZ-RhoGEF, LARG), and Rap1 GAP activity. The context-dependent outcome reflects differential coupling to these effectors across cell types and ligand environments.

Plexin-B1 supports the biology of several solid tumors and can modify antitumor immunity in breast cancer, making the *SEMA4D*/Plexin-B1 pathway relevant to metastatic disease immunotherapy [61]. It has also been implicated in the growth of estrogen receptor-positive breast cancer cells [62]. In prostate cancer, the P1597L Plexin-B1 variant reduces Rap GAP activity and increases Ras signaling, thereby favoring metastasis [63]. Plexin-B1 and Plexin-B2 can also promote resistance to androgen-deprivation therapy by enhancing Ran-dependent nuclear import of the androgen receptor [64]. In osteosarcoma, Sema4D/Plexin-B1 activates the Pyk2-PI3K/AKT cascade and accelerates tumor progression [65]. Ovarian cancer cells use HNF1A-AS1 to relieve miR-214-mediated repression of the *SEMA4D*/PLEXIN-B1/TIAM/RAC pathway, which promotes tumor growth and may support M2 macrophage polarization [66]. Drug-resistant B16F10R melanoma cells likewise upregulate *SEMA4D*, Plexin-B1, and PD-L1 [67]. Pepinemab, a humanized *SEMA4D*-neutralizing antibody, prevents ligand engagement with high- and low-affinity receptors, increases cytotoxic T-cell infiltration, delays tumor expansion, and sustains rejection in mouse models [68].

Receptor tyrosine kinase (RTK) crosstalk represents an important layer of Plexin-B signaling. Plexin-B1 physically interacts with MET (the HGF receptor) in glioblastoma and prostate cancer, where Sema4D/Plexin-B1 and HGF/MET synergistically activate migratory pathways. Plexin-B3 directly stimulates MET signaling in TNBC, linking B3 expression to breast cancer stem cell specification and lung metastasis [21]. With respect to ErbB2/HER2, Plexin-B1 co-expression modulates ERBB2-dependent signaling in breast cancer, and *SEMA4D* blockade may indirectly affect HER2-positive tumors. These RTK–plexin interactions suggest that combinatorial targeting, including plexin inhibition and RTK inhibition, may offer superior therapeutic efficacy.

Plexin-B2 is involved in glioblastoma, breast and ovarian cancers, and hematological malignancies via different mechanisms. In glioblastoma, the angiogenin/Plexin-B2 axis enhances invasion, vascular association, proliferation, and survival, and its blockade holds therapeutic promise [69]. The receptor decreases glioblastoma cell adhesion and favors infiltration, whereas Plexin-B2-deficient glioma stem cells aggregate more readily [70]. Plexin-B2 expressed by tumor-associated macrophages organizes cellular alignment and extracellular matrix architecture within invasive niches; deleting the receptor in these macrophages converts glioblastoma from an infiltrative to a more circumscribed growth pattern [14]. Tumor cells also need Plexin-B2 to regulate membrane tension, mechano-electrical coupling, and polarized migration in confined spaces [71]. Suppressing *PLXNB2* inhibits clustering and metastasis of circulating tumor cells in breast cancer [72]. The ligands Sema4C and Sema4A are involved in cluster formation and subsequent lung homing [72]. circ_0013958 in ovarian cancer acts via the miR-637/*PLXNB2* axis and may provide a therapeutic target [73]. High *PLXNB2* expression predicts unfavorable outcome in adult acute myeloid leukemia [74], potentially through a circ*PLXNB2*/miR-654-3p/*CCND1* regulatory circuit [75]. Plexin-B2 also promotes the anti-androgen resistance of prostate cancer, and *SEMA4D* blockade with pepinemab may affect both Plexin-B1 and Plexin-B2 signaling indirectly.

Plexin-B3 is strongly associated with triple-negative breast cancer and regulates hypoxia-responsive signaling, cell motility and invasion, and cancer stem cell differentiation. An experimental reduction in Plexin-B3 in mouse breast cancer models inhibits both primary tumor growth and metastatic spread [21]. Glycoproteomic analysis identified the receptor as a plentiful, accessible surface protein of triple-negative breast cancer cells. Depletion of Plexin-B3 thus reduces the proliferation, migration, and invasion of these cells [76]. However, this anti-tumor effect is context-dependent: in pancreatic cancer, Plexin-B3 overexpression promotes motility and invasion, functioning as a metastasis promoter rather than a tumor suppressor [59]. The hypoxia-dependent anti-tumor observation in TNBC (1% O_2_ in vitro; mouse xenograft models) [21] should therefore not be generalized across cancer types. The molecular basis for this duality—whether reflecting oxygen tension-dependent effector coupling or cell line-specific signaling—remains unresolved.

The three Plexin-B receptors thus have different oncogenic roles. Plexin-B1 is most closely associated with immune and hormone-dependent signaling, Plexin-B2 with glioma invasion and metastatic-cell clustering, and Plexin-B3 with hypoxic adaptation and triple-negative breast cancer. These differences are also present in individual cancers: breast cancer uses all three receptors in different ways, glioblastoma relies mainly on Plexin-B2, and prostate cancer can be divided into two types, Plexin-B1 and Plexin-B2, to evade treatment. The two receptors are regulated by different pathways in ovarian cancer. *SEMA4D* is shared by Plexin-B1 and Plexin-B2, so its blockade with pepinemab will inhibit both signaling pathways and strengthen anti-tumor immunity.

A major puzzle is the conflicting role of Plexin-B3 as a promoter of metastasis in pancreatic cancer [59] and a suppressor of growth and invasion in triple-negative breast cancer under hypoxia [21], either by oxygen tension-dependent signaling or cell-line-specific effector coupling, which is yet to be reconciled. Similarly, Plexin-B2 knockdown in rhabdomyosarcoma enhances tumor growth and reduces migration [60], contradicting the simple idea of Plexin-B2 as an always-pro-metastatic factor. The generalizability of the efficacy of pepinemab beyond Sema4D-high tumors also needs to be proven.

**Plexin B protein in Immune-Related Disease Conditions**

Plexin-B1 contributes to T-cell control in allergic airway disease and can limit asthma-associated inflammation [77]. *SEMA4A* binding to Plexin-B1 supports regulatory T-cell stability [39]. In allergic rhinitis, combined fexofenadine and fluticasone propionate lowers the expression of *SEMA4A*, *SEMA4C*, *SEMA4D*, *Plexin-B2*, and *Plexin-D1*, whereas budesonide plus fexofenadine increases *SEMA4A*, *SEMA4C*, *Plexin-B2*, and *Plexin-D1* transcripts while improving symptoms [78]. Plexin-B2 abundance in psoriatic tissue correlates with clinical severity [79]. MMP2-high fibroblasts use the CD100/Plexin-B2 axis to retain CD8+ T cells in skin and amplify psoriatic inflammation [80]. Keratinocytes also express Plexin-B2, the receptor for CD100, and increase its expression after exposure to contact allergens [81].

Plexin-B signaling is also implicated in intestinal and allergic immune disease. Semaphorin and plexin transcripts are increased in inflammatory bowel disease, particularly ulcerative colitis, consistent with an immunoregulatory role; *PLXNB1* expression is higher in affected patients than in healthy controls [82]. In children with atopic dermatitis, dibutyl phthalate exposure is associated with enrichment of the semaphorin–plexin and IL-5 pathways. Within this setting, Plexin-B1 correlates negatively with IL-10 and interferon-γ but positively with egg white-specific IgE [83]. Investigators have additionally created Fc-based agonistic and antagonistic Plexin-B1 dimerizers by fusing receptor-binding peptides to the Fc region of human IgG1, providing experimental tools for controlled pathway activation or inhibition [84]. Together, these observations connect Plexin-B1 with inflammatory bowel disease, ulcerative colitis, and atopic dermatitis through cytokine regulation and allergen-specific immune responses.

Therapeutic targeting of Plexin-B receptors requires careful assessment of target selectivity. In normal tissues, *PLXNB1* is expressed in astrocytes, vascular endothelium, and immune cells; *PLXNB2* in neural progenitors, macrophages, and bone marrow stroma; and *PLXNB3* in OPCs and retinal ganglion cells. In disease contexts, these patterns shift: *PLXNB2* is upregulated in glioblastoma invasive niches and breast cancer CTCs. *PLXNB1* is elevated in ulcerative colitis and PS19 tauopathy astrocytes. *PLXNB3* is overexpressed in pancreatic cancer. The differential expression between normal and disease tissues provides a therapeutic window, but systemic inhibition carries risks of disrupting physiological functions.

Plexin-B2 is involved in allergic and autoimmune inflammation, in skeletal aging, in intestinal epithelial immunity, and in asthma. Grancalcin is released by senescent immune cells, binds Plexin-B2 on bone marrow mesenchymal stem cells, suppresses osteogenesis, and promotes their adipogenic differentiation. Deleting Plxnb2 in skeletal stem cells impairs amelioration of the aged-bone phenotype [85]. In the intestine, AP-1B controls the proper trafficking of regulatory proteins required to maintain intraepithelial lymphocytes, establishing a Plexin-B2-dependent aspect of epithelial immune homeostasis [86]. Airway epithelial cell exosomes carrying *PLXNB2* can worsen allergic inflammation by promoting MMP14-dependent CD100 shedding and macrophage activation, suggesting a target for preventing asthma exacerbations [87]. Primary Sjögren’s syndrome is associated with increased CX3CR1 on cDC2 cells but reduced integrin β2 and Plexin-B2 expression [88]. In bisphosphonate-related osteonecrosis of the jaw, activated γδ T cells release MMP3, which cleaves membrane Sema4D to generate a soluble ligand. Soluble Sema4D then binds Plexin-B1/2 on osteoblasts, activates mTOR signaling, downregulates ALP and Runx2, and suppresses osteogenic differentiation [89]. These mechanisms position Plexin-B2 at several immune–stromal interfaces involving bone, gut, lung, and exocrine autoimmunity.

Plexin-B1 and Plexin-B2 thus have partially different immune associations. Plexin-B1 is highly expressed in inflammatory bowel disease and atopic dermatitis, and Plexin-B2 has also been associated with skeletal aging, asthma, Sjögren’s syndrome, and intestinal immune regulation multiple times. Although few studies have directly compared both receptors in the same disease, the joint data have shown that B-class plexins are required for communication among immune, epithelial, and stromal cells.

Plexin-B1 is elevated in ulcerative colitis [82] but shows negative correlation with anti-inflammatory cytokines IL-10 and IFN-γ in atopic dermatitis [83], raising the question of whether its immune role is tissue-specific or stage-dependent. The CD100–Plexin-B2 axis appears to exert opposing effects in psoriasis (promoting CD8^+^ residency [80]) versus asthma (exosome-mediated inflammation [87]), implying ligand-context duality. How Plexin-B2 balances intestinal homeostasis versus autoimmune flare remains an open question.

**Plexin B protein in cardiovascular disease ailments**

Plexin-B1 is a typical Sema4D receptor that is involved in vascular calcification, angiogenesis, stem cell differentiation, and metastasis. Both Sema4D and Plexin-B1 are elevated in calcified aortas from rats with chronic kidney disease and in phosphate-induced calcification of vascular smooth-muscle cells; genetic disruption of either component alters smooth-muscle phenotype and apoptosis [90]. Sema4D/Plexin-B1 signaling in vascular repair induces METTL3-dependent secretion of PDGF-BB to recruit dental-pulp stem cells and stabilize new blood vessels [91]. The same receptor pathway also promotes vasculogenic differentiation of dental pulp-derived stem cells [92]. NEAT1 expression in ovarian cancer is associated with angiogenesis factors, such as Sema4D and Plexin-B1, and is thus involved in tumor vascularization and metastasis [93]. Sema4D enhances the angiogenic effect by increasing PDGF-BB and activating Plexin-B1 on endothelial cells [94]. As Sema4D can signal via Plexin-B1, Plexin-B2, and CD72, it is likely that the vascular and pathological effects will spread beyond a single receptor [95].

Plexin-B2 also regulates vascular biology in a way that does not require Sema4D. Angiogenin sustains endothelial proliferation by binding *PLXNB2* and stimulating ribosomal RNA transcription [96]. Although Sema4D is capable of binding Plexin-B2, no research has directly linked this receptor–ligand pair to vascular calcification or angiogenesis to date.

Plexin-B1 and Plexin-B2 thus contribute differently to the vascular system. Plexin-B1 mainly mediates Sema4D-dependent pathological and reparative responses, Plexin-B2 supports endothelial homeostasis through angiogenin-dependent proliferation, and Plexin-B3 has not shown a similar function. RNA sequencing analysis of diabetic bone-implant models shows that the TGF-β, NF-κB/MAPK, Wnt, and TNF pathways are all altered, and guidance and adhesion signals between endothelial cells and osteoblasts are disrupted. The modifications have reduced angiogenesis–osteogenesis coupling and are therefore linked to NOX2-induced oxidative stress upstream of the vascular defect [97]. While Plexin-B1 clearly mediates Sema4D-driven vascular calcification [90], the contribution of Plexin-B2, which also binds Sema4D [91], to this process is surprisingly underexplored. Additionally, the angiogenin–Plexin-B2 axis supports endothelial homeostasis [96] yet is co-opted by glioblastoma for invasion [69], highlighting a context-dependent switch between physiological and pathological angiogenesis. The cardiovascular functions of Plexin-B3 remain almost entirely unknown and represent a critical gap.

**Biomarker-guided patient selection**

Given the context-dependent nature of Plexin-B signaling, biomarker-guided patient selection will be critical for clinical translation. Candidate biomarkers include Plexin-B expression levels—*PLXNB2* is a prognostic marker in AML and correlates with CTC clustering in breast cancer; semaphorin ligand levels—*SEMA4D* immunohistochemistry may identify tumors reliant on Sema4D/Plexin-B1 signaling; downstream activity—RhoA/Rac1 activation status could serve as a pharmacodynamic readout; and receptor-RTK co-expression—Plexin-B3/MET co-expression in TNBC may identify responsive tumors. A combinatorial biomarker panel incorporating these parameters would maximize the therapeutic index of plexin-targeted interventions.

**Potential adverse effects of systemic Plexin-B inhibition**

Systemic inhibition of Plexin-B receptors carries potential adverse effects that must be considered. Plexin-B2 is essential for cortical development and neural differentiation, its inhibition during development could disrupt brain architecture. The angiogenin–Plexin-B2 axis supports endothelial homeostasis and vascular repair; chronic blockade may impair wound healing or vascular regeneration. Plexin-B1/B2 regulate immune cell positioning in the intestine and skin; systemic inhibition could disrupt barrier immunity or exacerbate inflammatory conditions. Plexin-B receptors maintain tissue homeostasis in bone, liver, and pancreas. These considerations argue for tissue-specific or temporally restricted targeting strategies rather than continuous systemic suppression.

**Plexin-B protein in other diseases issues**

Research into plexin family proteins has identified Plexin-B1 and B2, alongside their molecular regulation, pathological role, and therapeutic potential. The contribution of TMEM260-mediated O-mannose sylation to Plexin-B2 maturation [23] and the allosteric regulatory mechanism of Plexin-B1 in the transmission of signals [24] has been studied. Peripheral *PLXNB2* is shown to be related to the risk of diabetic retinopathy [25], and chromatin conformation changes in NAFLD were found to be related to genomic rearrangements at the Plxnb1 locus [26]. Anti-Plexin-B1 antibody has shown promise in osteoporosis and multiple sclerosis models, indicating its therapeutic value [28].

The Sema4D and Plexin-B1 signaling axis significantly influences bone formation, osteoarthritis, and wound healing. Metformin can counteract Sema4D’s promotion of bone formation by reducing Plexin-B1 expression, while Exendin-4 hinders Sema4D by activating CRMP2 [98]. Elevated Sema4D in early TMJ OA reduces osteogenic activity by binding to Plexin-B1 on osteoblasts [99]. Sema4D also impedes IGF-1-induced osteogenesis by binding to Plexin-B1 on osteoblasts [100]. In diabetic wound healing, exosomes inhibit Plexin-B1 of Sema4D, enhancing the osteogenic differentiation of stem cells and wound healing [101]. Aberrant Plexin-B1 expression in testicular tissue correlates with increased pro-apoptotic markers, potentially impacting male reproductive health [102]. Plexin-B1 positive nerve cells in the patellar tendon increased [42]. Mir-362-5p promotes osteoarthritis by inhibiting *PLXNB1* [103]. Sema4D/Plexin-B1 signaling perturbation affects gonadotropin-releasing hormone development in mice, highlighting Plexin-B1 mutations in IHH [104]. CD100 (Sema4D) regulates gene expression in PHH via Plexin-B1 and Plexin-B2 signaling, activating ERK and STAT3 pathways [105]. *Plexin-B1* conditional gene knockout mice were created using CRISPR/Cas9 technology, confirming *Plexin-B1*’s role [106].

Research indicates that Plexin-B1 possesses GAP function and cooperates with RAP against Rap1b through its N-terminal near-membrane region. A new loop prevents the access of Rap1b to the Plexin-B1 GAP domain [107]. Without Rap1b, Rnd1 binds to Plexin-B1 more strongly and stably than Rac1 [108]. An autonomous mechanochemical feedback of Plexin-B that is involved in inhibiting tumor growth in basal cell carcinoma highlights the importance of Plexin-B as a mechanical sensor and couples density sensing with cell division [109]. Although not specific to Plexin-B1, this study indicates the general importance of Class B Plexin proteins in mechanical transduction.

Plexin-B2 is also involved in multiple domains like genetic diseases, diabetes, cellular aging, mechanical sensing, and intestinal health. Plexin-B2 is involved in a genetic syndrome of enamel defects and hearing loss caused by mutations in the *PLXNB2* gene [110]. In diabetic nephropathy, *PLXNB2* and VAP-1 are highly expressed [111]. Plexin-B2 is associated with diabetes risk [112]. Grancalcin-induced Plexin-B2 activation causes cellular senescence through ARG2-mediated mitochondrial dysfunction [113]. In mechanical responses, Plexin-B2 contributes to tension-induced bone formation via the RhoA pathway, F-actin alignment, and YAP translocation [114]. In temporomandibular joint osteoarthritis, ECM stiffening triggers the Plexin-B2/YAP/SLIT3 axis to promote angiogenesis and cartilage degradation [115]. Plexin-B2 plays a key role in regulating cytoskeletal tension, cell adhesion, and cellular mechanics in stem cell physiology and tissue development [38]. Loss of AP-1B in intestinal epithelial cells reduces basolateral membrane proteins, including Plexin-B2 [86]. Sema4D can induce ERK and STAT3 pathways through Plexin-B2 to regulate gene expression in primary human hepatocytes [105].

Plexin-B1 and Plexin-B2 have unique functions in bone, metabolism, mechanical signaling, and immunity. At the same time, some of their pathways in the downstream signaling of Sema4D are overlapping. All of these results highlight the significant physiological and pathological relevance of the Plexin-B family.

**Concluding remarks**

The Plexin-B family acts as a context-sensitive signaling hub in human disease. Recent studies have linked aberrant Plexin-B pathways to neurodegeneration [116,117], autoimmunity, malignancy, and cardiovascular pathology, but the direction and consequences of signaling are receptor-, ligand-, and cellular context-dependent (Table 3). Of the plexin-related targets, the following are most relevant to further development: (1) *SEMA4D*-neutralizing antibodies (e.g., pepinemab) are the most clinically advanced, with phase I/II trials ongoing in recurrent solid tumors; (2) antagonistic anti-Plexin-B1 monoclonal antibodies have preclinical efficacy in postmenopausal osteoporosis and multiple sclerosis and are promising precision medicine candidates; (3) *PLXNB2*-targeted silencing or inhibition has promise in glioblastoma and metastatic breast cancer but must be managed for off-target effects; (4) Plexin-B3 modulation is still in the preclinical phase, and context-specific—as well as hypoxia-dependent—approaches are required. Unifying information from these fields, this review elucidates how individual Plexin-B members may have divergent effects and critically evaluates their potential therapeutic value. In the future, it will be important to delineate receptor-specific signaling networks with higher spatial and temporal resolution, to discard compensatory reactions in favor of causal mechanisms, and to determine which disease contexts are most amenable to plexin-directed intervention.

## Figures and Tables

**Table 1:** Physiological and pathophysiological effects of Plexin-B family members.

Receptor	Physiological Role	Pathological Role	Disease Context
Plexin-B1	Axon guidance; astrocyte morphology; vascular repair	Neuroinflammation; tumor promotion; vascular calcification	Alzheimer’s, glioblastoma, prostate cancer, CKD
Plexin-B2	Cortical tension; GABAergic synapses; Schwann cell positioning	Glioblastoma invasion; CTC clustering; psoriasis	GBM, breast cancer, AML, schizophrenia
Plexin-B3	Oligodendrocyte maturation; retinal axon crossing	Amyloid-β secretion; TNBC growth; pancreatic metastasis	Alzheimer’s, TNBC, pancreatic cancer

**Table 2:** Comparative overview of Plexin-B1, B2, and B3.

Receptor	Major Ligands	Principal Downstream Pathways	Cancer Roles	Immune/Neural Roles	Therapeutic Opportunities
B1	*SEMA4D*, *SEMA4A*	R-Ras GAP; RhoA/ROCK; Pyk2-PI3K/AKT	prostate, GBM, osteosarcoma	Astrocyte–microglia spacing; AD; synapse assembly	Antibody/small-molecule approaches
B2	*SEMA4C*, *SEMA4B*, angiogenin	RhoA/ROCK; ID1/3; Rac1	GBM invasion; CTC clustering; AML	Cortical tension; GABA synapses; schizophrenia	Targeted inhibition
B3	HSPA5; context-dependent Sema	MET; hypoxia signaling	TNBC (suppressor), pancreatic (promoter)	OPC maturation; retinal axon guidance; AD	Context-specific modulation

**Table 3:** Plexin-B signaling across diseases: models and evidence levels.

Receptor	Disease/Model	Biological Effect	Molecular Mechanism	Evidence Level	Ref.
B1	AD (mouse, human tissue)	Astrocyte–microglia spacing	Peri-plaque glial net organization	In vivo/patient sample	[35,37]
B1	Prostate cancer (mouse model)	Metastasis promotion	Rap GAP	Transgenic mouse	[58,63]
B1	Osteosarcoma (cell culture)	Tumor progression	Pyk2-PI3K/AKT	Cell line	[65]
B2	GBM (PDX, mouse)	Invasion, vascular association	RhoA; membrane tension	PDX/mouse model	[14,69,70,71]
B2	Breast cancer (mouse)	CTC clustering; metastasis	Sema4C/Sema4A to Rac1	Mouse model/clinical dataset	[72]
B2	AML (patient samples)	Unfavorable prognosis	circ*PLXNB2*/miR-654-3p/*CCND1*	Patient IHC/TCGA	[74,75]
B3	TNBC (mouse, in vitro)	Growth and metastasis suppression	Hypoxia-responsive; MET signaling	Mouse xenograft/glycoproteomics	[21,76]
B3	Pancreatic cancer (cell culture)	Motility and invasion	Actin reorganization	Cell line	[59]
B2	Psoriasis (human tissue)	CD8+ T-cell retention	CD100/Plexin-B2 to regulate MMP2	Patient biopsy	[79,80]
B1	Inflammatory bowel disease	Immunoregulation	*SEMA4D*–Plexin-B1 axis	Patient cohort	[82]

## Data Availability

Data in this publication will be made available on request.
